# Interventional radiology in woman’s health: room for improvement

**DOI:** 10.1186/s42155-023-00376-9

**Published:** 2023-05-17

**Authors:** Elika Kashef, Maria Tsitskari

**Affiliations:** 1 Imperial College Healthcare NHS Trust, London, UK; 2 Mediterranean Hospital, Limassol, Cyprus

It is with great pleasure for us to present to you this exciting edition of CVIR Endovascular with a focus on women’s health.

The two-tier system of healthcare for males vs females, resulting in health inequities affecting individual health seeking behaviour, poorer outcomes and delayed diagnosis, has long been recognised. This is a complex topic and the root cause of it is multifaceted, however in the 1960’s following the thalidomide scandal, there was a ban on pregnant women as well as women of child bearing age to partake in any trials. Although this was aimed to protect women, what it did end up doing was to side line half the population from important clinical trials which has led to poor recognition of disease patterns and worse outcomes for the female population.

Caroline Criado Perez, in her book, ‘Invisible Women: Exposing Data Bias in a World Designed for Men’, [1] has gathered various statistics that show how women are treated unevenly. She has argued that the data shows that healthcare is “systematically discriminating against women, leaving them chronically misunderstood, mistreated and misdiagnosed”.

Gender bias and discrimination occurs at many levels throughout the healthcare system, from the interactions between patients and doctors to the medical research and policies that govern it. In 2018 a study on patients with chronic pain concluded that doctors often view men with chronic pain as “brave” or “stoic,” but women as “emotional” or “hysterical.” The study also found that women’s pain was more likely to be treated as a symptom of a mental health condition, rather than a symptom of a physical condition [2].

Less is also known about conditions that only affect women, including common gynaecological conditions that can have severe impacts on health and wellbeing. As an example, we now know that pelvic pain is poorly understood and women with pelvic pain have been under-diagnosed and at times dismissed for decades. Endometriosis was once considered a condition that occurs in women in their mid-30’s but we now see the disease pattern emerging in younger age groups due to the increased utilisation of imaging.

Luckily, we have moved forward from that period and since 1993 women have been included in trials and studies globally.

As a result, we have emerging data on all aspects of pathologies that effect men and women, but also conditions that are specific to the female cohort.

In this edition we focus on a few key conditions affecting women.

Drs Mailli and Ratnam will review the evidence for uterine artery embolization focusing on three challenging aspects—post procedure fertility, symptomatic adenomyosis and large volume fibroids and uteri which remains an important topic [3].

Prof Hemingway, who published the first ever English language paper on ovarian vein embolization, will look through the journey of treatments available for pelvic congestion syndrome, a disease that is often undiagnosed in women with pelvic pain [4]. The lack of diagnosis seems to be related to limited awareness by both physicians and patients, in combination with limited data in the literature.

Dr Roberts, will discuss fallopian tube recanalization techniques and outcomes [5] and Dr O’Sullivan will finish the edition with review on deep venous stenting in women [6].

These topics are all equally important and many have been under recognised for decades. Pelvic congestion syndrome remains a condition that some clinicians do not believe in and never get treatment. Many patients have to undergo laparoscopy for infertility prior to consideration for fallopian recanalization and in many countries fibroid embolization is still not offered to patients as of one of their treatment optional when discussing invasive interventions.

This edition will highlight how far we have come in these common conditions and this will hopefully not only demonstrate an exciting overview of results and outcomes to date, but also to inspire and motivate our younger interventional radiologists to instigate and contribute to new trials and carry the banner for the next generation.

For generations women despite making up 50% of the population have lived with a healthcare system that was designed by men, for men. In this postpandemic era we should work together to elevate the importance of women’s health across the entire health industry. Women participation in studies should always be analysed also as a separate group and differences in outcome between men and women should be taken seriously. Also, more studies focusing on women health issues should be undertaken. It is for instance now clear that coronary disease in women is sometimes completely different as in men. The lessons we have learned have the power to transform the future of healthcare and ultimate, enable better care for women worldwide.

## Data Availability

Not applicable.
